# Supplementary material for: Multi‐dataset identification of innovative feature genes and molecular mechanisms in keratoconus
Source: J Cell Mol Med. 2024 Sep 19;28(18):e70079. doi: 10.1111/jcmm.70079 (PMC11412914; doi:10.1111/jcmm.70079)
Supplement: Supplementary file 2 — Material S2. [file JCMM-28-e70079-s002.zip › 7.GSEA analysis/1.ARL11/KEGG_PYRUVATE_METABOLISM.html]

Details for gene set KEGG\_PYRUVATE\_METABOLISM[GSEA]

|  || Dataset | ARL11.group.cls#h\_versus\_l |
| Phenotype | group.cls#h\_versus\_l |
| Upregulated in class | l |
| GeneSet | KEGG\_PYRUVATE\_METABOLISM |
| Enrichment Score (ES) | -0.26378325 |
| Normalized Enrichment Score (NES) | -1.0301129 |
| Nominal p-value | 0.4375 |
| FDR q-value | 1.0 |
| FWER p-Value | 0.999 |
Table: GSEA Results Summary

  

Fig 1: Enrichment plot: KEGG\_PYRUVATE\_METABOLISM      
 Profile of the Running ES Score & Positions of GeneSet Members on the Rank Ordered List

  

| SYMBOL | TITLE | RANK IN GENE LIST | RANK METRIC SCORE | RUNNING ES | CORE ENRICHMENT || 1 | ACACB | na | 5077 | 0.163 | -0.0887 | No |
| 2 | PCK1 | na | 5840 | 0.141 | -0.0609 | No |
| 3 | LDHAL6A | na | 6578 | 0.122 | -0.0390 | No |
| 4 | ACSS1 | na | 6583 | 0.122 | 0.0039 | No |
| 5 | DLD | na | 9166 | 0.072 | -0.0450 | No |
| 6 | ACOT12 | na | 9407 | 0.069 | -0.0277 | No |
| 7 | ACACA | na | 9664 | 0.065 | -0.0122 | No |
| 8 | ME3 | na | 10011 | 0.060 | -0.0010 | No |
| 9 | LDHD | na | 10131 | 0.059 | 0.0164 | No |
| 10 | ALDH7A1 | na | 10913 | 0.048 | 0.0109 | No |
| 11 | PDHA2 | na | 12632 | 0.030 | -0.0280 | No |
| 12 | ALDH3A2 | na | 13283 | 0.025 | -0.0380 | No |
| 13 | PC | na | 13365 | 0.024 | -0.0319 | No |
| 14 | LDHC | na | 13378 | 0.024 | -0.0238 | No |
| 15 | ME2 | na | 14156 | 0.018 | -0.0400 | No |
| 16 | PDHB | na | 15297 | 0.009 | -0.0696 | No |
| 17 | MDH2 | na | 16386 | 0.001 | -0.1006 | No |
| 18 | ACSS2 | na | 16411 | 0.001 | -0.1010 | No |
| 19 | PDHA1 | na | 17379 | -0.006 | -0.1268 | No |
| 20 | HAGH | na | 17778 | -0.009 | -0.1352 | No |
| 21 | ACYP2 | na | 18129 | -0.011 | -0.1413 | No |
| 22 | GRHPR | na | 18147 | -0.011 | -0.1377 | No |
| 23 | PKM | na | 18421 | -0.013 | -0.1410 | No |
| 24 | MDH1 | na | 18516 | -0.014 | -0.1388 | No |
| 25 | HAGHL | na | 19756 | -0.022 | -0.1668 | No |
| 26 | ACAT2 | na | 20041 | -0.024 | -0.1665 | No |
| 27 | PKLR | na | 21593 | -0.036 | -0.1985 | No |
| 28 | LDHB | na | 22665 | -0.047 | -0.2129 | Yes |
| 29 | ACAT1 | na | 22983 | -0.050 | -0.2044 | Yes |
| 30 | DLAT | na | 22996 | -0.050 | -0.1871 | Yes |
| 31 | GLO1 | na | 24571 | -0.070 | -0.2077 | Yes |
| 32 | LDHA | na | 24699 | -0.072 | -0.1859 | Yes |
| 33 | ALDH1B1 | na | 25126 | -0.078 | -0.1707 | Yes |
| 34 | ALDH9A1 | na | 25157 | -0.078 | -0.1440 | Yes |
| 35 | ACYP1 | na | 26274 | -0.095 | -0.1428 | Yes |
| 36 | PCK2 | na | 30472 | -0.203 | -0.1921 | Yes |
| 37 | AKR1B1 | na | 30604 | -0.208 | -0.1226 | Yes |
| 38 | LDHAL6B | na | 31068 | -0.223 | -0.0574 | Yes |
| 39 | ALDH2 | na | 31150 | -0.226 | 0.0200 | Yes |
| 40 | ME1 | na | 31396 | -0.235 | 0.0961 | Yes |
Table: GSEA details [plain text format]

  

Fig 2: KEGG\_PYRUVATE\_METABOLISM      
 Blue-Pink O' Gram in the Space of the Analyzed GeneSet

  

Fig 3: KEGG\_PYRUVATE\_METABOLISM: Random ES distribution      
 Gene set null distribution of ES for **KEGG\_PYRUVATE\_METABOLISM**
